# Multifactorial Machine Learning Algorithm Integration of Pain Mechanisms Can Predict the Efficacy of 3‐Week NSAID Plus Paracetamol in Patients With Painful Knee Osteoarthritis

**DOI:** 10.1002/ejp.70140

**Published:** 2025-09-29

**Authors:** Rocco Giordano, Lars Arendt‐Nielsen, Emma Hertel, Anne Estrup Olesen, Kristian Kjær‐Staal Petersen

**Affiliations:** ^1^ Center for Neuroplasticity and Pain (CNAP), Department of Health Science and Technology Aalborg University Aalborg Denmark; ^2^ Center for Mathematical Modeling of Knee Osteoarthritis (MathKOA), Department of Material and Production, Faculty of Engineering and Science Aalborg University Aalborg Denmark; ^3^ Department of Gastroenterology & Hepatology, MechSense Aalborg University Hospital Aalborg Denmark; ^4^ Steno Diabetes Center North Denmark Aalborg University Hospital Aalborg Denmark; ^5^ Department of Clinical Medicine Aalborg University Aalborg Denmark; ^6^ Department of Clinical Pharmacology Aalborg University Hospital Aalborg Denmark

## Abstract

**Background:**

Studies demonstrate that pain sensitization, epigenetic mechanisms, inflammation, and psychological factors might be predictive of treatment outcomes. Anti‐inflammatory therapy is recommended, but efficacy varies among patients. This study aimed to utilise machine learning to predict the analgesic responses of 3‐week NSAID plus paracetamol therapy using pre‐treatment assessments of pain sensitivity, inflammation, microRNA, and psychological factors.

**Methods:**

Patients (*n* = 101) underwent 3‐week combined NSAID plus paracetamol therapy. Pain sensitivity using cuff algometry, Hospital Anxiety and Depression Scale, Pain Catastrophizing Scale, EQ‐5D‐3L scale, and blood samples were collected before therapy. Pain relief was assessed by the Knee Injury and Osteoarthritis Outcome Score pain subscale, before and after therapy. Inflammatory biomarkers were analysed using Olink, and microRNA using Next‐Generation RNA Sequencing. Data Integration Analysis for Biomarker discovery using Latent cOmponents (DIABLO) was utilised to integrate the pre‐treatment data and explain the analgesic effect.

**Results:**

DIABLO model identified 30 significant variables across the 4 domains. After cross‐validation, model performance showed an area under the precision‐recall curve of 85%, sensitivity of 83%, specificity of 87%, and balanced accuracy of 85%.

**Conclusions:**

This study utilises a machine learning algorithm, based on pain sensitization, epigenetics, inflammatory response, and psychological factors, to predict analgesic response in osteoarthritis patients. The study demonstrates that incorporating multiple factors into a model enhances its performance, enabling the identification of patients who will benefit from therapy, advancing personalised pain management.

**Significance Statement:**

In this study, a machine learning algorithm, based on pain sensitization, epigenetic mechanisms, inflammatory response, and psychological factors, predicts analgesic response in osteoarthritis patients with 84% accuracy.

**Trial Registration:**

ClinicalTrials.gov identifier: NCT02967744

## 1 Introduction

Knee osteoarthritis (OA) is a prevalent degenerative joint disease that impairs the quality of life for millions worldwide (Litwic et al. 2013). Chronic pain is the hallmark symptom of OA, but the severity of pain does not correlate with structural damage, indicating that other factors contribute to pain in OA (Neogi 2013). Studies have found that multiple, individual factors can impact pain in OA, such as pain sensitization, epigenetic mechanisms, inflammatory responses, and psychological factors (Giordano, Capriotti, et al. 2024; Hertel et al. 2024; Kurien et al. 2022; Petersen et al. 2016; Schaible 2014).

Quantitative sensory testing (QST) can be used to assess neurological components of pain in OA and has previously been demonstrated to be associated with clinical pain intensity (Arendt‐Nielsen, Egsgaard, et al. 2015) and demonstrated the ability to predict the analgesic effect of standard pain therapy in OA (Georgopoulos et al. 2019; Petersen et al. 2022). Systemic low‐grade inflammation has been associated with pain intensity in knee OA (Robinson et al. 2016), and the preoperative inflammatory status might also be linked to the risk of chronic postoperative pain after total knee replacement surgery (Gandhi et al. 2013; Giordano, Ghafouri, et al. 2024). Around 20% of OA patients exhibit symptoms of anxiety, depression, or pain catastrophizing (Stubbs et al. 2016), and higher levels of these are associated with higher clinical pain in OA and a poorer response to standard pain therapy (Edwards et al. 2016; Hertel et al. 2024). Additionally, among epigenetic changes, specific microRNAs (miRNAs), such as miR‐146a‐5p and miR‐183, have been demonstrated to be correlated with clinical pain (Bali et al. 2021; Giordano et al. 2020; Reinhold et al. 2023).

Some of the mechanisms might be interconnected, as demonstrated by, for example, associations between inflammation and psychological factors (Giordano, Capriotti, et al. 2024), miRNAs and inflammation (Bali et al. 2021; Giordano et al. 2020), and QST and psychological factors (Mansfield et al. 2024). Recent data suggest that the integration of several mechanisms provides a stronger explanation of clinical pain in OA than only assessing a single domain (Hertel et al. 2024). In a cross‐sectional study, QST, systemic inflammation, psychological factors, and miRNA alterations have been shown to form interconnected pain mechanistic networks (Giordano, Arendt‐Nielsen, et al. 2024), but no studies have investigated if these mechanistic networks can predict a treatment response.

The second‐line treatment for pain in OA often includes non‐steroidal anti‐inflammatory drugs (NSAIDs) alone or in combination with other drugs (Hochberg et al. 2012), but the analgesic effect varies (Hochberg et al. 2012). As NSAIDs administration can be associated with a variety of adverse events (da Costa et al. 2017) it is, therefore, crucial to quickly identify patients who are likely to benefit from the therapy. This targeted approach helps avoid the need for patients to cycle through multiple treatments before getting the correct treatment.

The present work aims to utilize multifactorial machine learning modeling to predict the analgesic responses to 3 weeks of combined NSAID and paracetamol therapy using pre‐treatment assessments of pain sensitivity, inflammation markers, microRNAs, and psychological factors.

## 2 Materials and Methods

### 2.1 Patients' Cohort

Patients were recruited in the period between January 2016 and February 2018 at the Orthopaedic Outpatient Clinic at Aalborg University Hospital, Aalborg, Denmark. The presence of clinical OA was defined based on the American College of Rheumatology criteria. Exclusion criteria involved the presence of gastrointestinal ulcers, asthma, or other allergic reactions to NSAIDs, kidney disease, myocardial infarction within the past 6 months, severe hypertension (defined as systolic ≥ 180 mmHg, diastolic ≥ 110 mmHg), severe thrombocytopenia (thrombocyte count < 50 × 10^9^/L), fibromyalgia diagnosis or widespread pain, and insufficient liver or kidney function.

### 2.2 Study Design

The current report is a secondary analysis of data collected as a part of a study investigating the effect of a 3‐week treatment with NSAIDs and paracetamol (Petersen, Olesen, et al. 2019). The assessments and baseline pain outcome used in the current study were collected before any treatment commenced, while the follow‐up pain outcome was collected at the end of treatment (i.e., 3 weeks after initiating the combined NSAID and paracetamol treatment). The study was approved by the North Denmark Region Committee on Health Research Ethics, Denmark (N‐20140077) and was registered at ClinicalTrials.gov.

### 2.3 Treatment

Patients received 400 mg ibuprofen three times daily (*ter in die* (TID)) and 1 g paracetamol TID for 3 weeks. In addition, 20 mg of pantoprazole was administered once daily to prevent gastrointestinal ulcers. All patients were instructed to report any adverse events.

### 2.4 Pain Assessment

The Knee Injury and Osteoarthritis Outcome Score (KOOS) is a frequently used questionnaire capturing patient‐reported outcomes in people with knee injuries and knee OA. The questionnaire contains 5 subscales: pain (9 items), activities of daily living (17 items), sport and recreation function (5 items), knee‐related quality of life (4 items), and other symptoms (7 items). The subscales are each scored from zero to 100, corresponding to extreme knee problems and no knee problems, respectively (Collins et al. 2016). In the present study, the KOOS pain subscale was the only subscale included, and it was used as the main indicator for OA‐related knee pain.

### 2.5 Patients Reported Outcomes

#### 2.5.1 The Hospital Anxiety and Depression Scale

The Hospital Anxiety and Depression Scale (HADS) consists of 14 items with two 7‐item subscales measuring anxiety (HADS_A_) and depression (HADS_D_) symptoms, respectively. Within both subscales, higher scores denote more severe symptoms. Each item is scored from 0 to 3, giving a total subscale score range of 0 to 21 for both anxiety and depression. Within both subscales, higher scores indicate more severe symptoms. The HADS has been found valid in assessing symptoms of anxiety and depression, as well as symptom severity (Bjelland et al. 2002; Christensen et al. 2020). In this study, the aggregate scores for both the anxiety and depression subscales of the HADS were utilised for analysis.

#### 2.5.2 The Pain Catastrophizing Scale

Pain catastrophizing is based on the earlier conceptualizations of catastrophizing found in patients with anxiety and depressive disorders, and it is defined as an exaggerated negative cognitive and emotional response to actual or anticipated pain (Quartana et al. 2009). The Pain Catastrophizing Scale consists of 13 items measuring pain‐related thoughts and feelings. Each item is rated on a 4‐point scale ranging from 0 to 4, corresponding to thinking/feeling that way during pain, “not at all” and “very much,” respectively. The Pain Catastrophizing Scale (PCS) is considered the most comprehensive assessment tool for the evaluation of negative cognitive status, and its three‐factor hierarchical structure consisting of magnification, helplessness, and rumination has been previously validated and demonstrated (Osman et al. 1997; Sullivan et al. 1995). In this study, the total scores from the PCS were utilized for analysis.

### 2.6 Health‐Related Quality of Life

Health‐related quality of life was captured using the 3‐level EuroQol 5‐dimensions EQ‐5D‐3L health EQ VAS anchored at 0, “The worst health you can imagine,” and 100, “The best health you can imagine” (Feng et al. 2021; Rabin and De Charro 2001). In this study, the overall Health‐Related Quality of Life (HRQoL) scores were utilized for analysis.

### 2.7 Quantitative Sensory Testing (QST)

The cuff algometer (Cortex Technology, Hadsund, Denmark, and Aalborg University, Aalborg, Denmark) was utilised to assess pressure pain thresholds (PPTs), temporal summation of pain (TSP), and conditioned pain modulation (CPM). The test–retest reliability of the cuff algometer is good‐to‐excellent (Graven‐Nielsen et al. 2015, 2017; Imai et al. 2016; Vaegter and Graven‐Nielsen 2016).

#### 2.7.1 Cuff Pressure Pain Thresholds

The pressure was increased at a rate of 1 kPa/s, and the patient was instructed to continuously rate their pain on the electronic visual analog scale until reaching their tolerance. The cuff pressure pain threshold (cPPT) was defined as the pressure at which the electronic visual analog scale exceeded 1 cm. Values of pressure pain threshold were used in the analysis.

#### 2.7.2 Temporal Summation of Pain

Ten repeated mechanical pressure stimuli were delivered at 0.5 Hz (1‐s stimuli duration and 1‐s interstimulus intervals). During the 10 repeated stimuli, the subjects continuously rated the pain intensity on the electronic VAS. TSP was used in the analysis and defined as the difference between the electronic visual analog scale scores of the tenth and first stimuli.

#### 2.7.3 Conditioned Modulation of Pain

An additional tourniquet cuff was fitted around the contralateral leg, and a painful tonic stimulus was applied at 70% of the pressure pain tolerance recorded previously. Simultaneously, the other cuff applied a gradually increasing pressure of 1 kPa/s, rated continuously on the electronic visual analog scale until tolerance was reached, indicated by the patient pressing the stop button. The CPM effect was defined as the difference between the PPT during and before the conditioned modulation of pain, with a positive value denoting functional CPM.

### 2.8 RNA‐Sequencing for microRNAs Analysis

RNA was isolated from 200 μL of plasma using the miRNeasy Serum/Plasma Kit (QIAGEN) according to the manufacturer's instructions with an elution volume of 14 μL. The library preparation was done using the QIAseq miRNA Library Kit (QIAGEN). A total of 5 μL total RNA was converted into miRNA NGS libraries. After adapter ligation, UMIs were introduced in the reverse transcription step. The cDNA was amplified using PCR (22 cycles), and during the PCR, indices were added. After PCR, the samples were purified. Library preparation was quality controlled using capillary electrophoresis (Fragment Analyser HS NGS Fragment Kit (1‐6000 bp)). Based on the quality of the inserts and the concentration measurements, the libraries were pooled in equimolar ratios. The library pool(s) were quantified using qPCR. The library pool(s) were then sequenced on a NextSeq (Illumina Inc.) sequencing instrument according to the manufacturer's instructions (1 × 75, 2 × 10). Raw data was de‐multiplexed, and FASTQ files for each sample were generated using the bcl2fastq2 software (Illumina Inc.). All primary analysis was carried out using CLC Genomics Server 23.0.5. The workflow “QIAseq miRNA Quantification” of CLC Genomics Server with standard parameters was used to map the reads to miRBase version 22. In short, the reads were processed by (1) trimming of the common sequence, UMI, and adapters, and (2) filtering of reads with a length < 15 nt or length > 55 nt. They were then deduplicated using their UMI. Reads were grouped into UMI groups when they (1) started at the same position based on the end of the read to which the UMI was ligated (i.e., Read2 for paired data), (2) were from the same strand, and (3) had identical UMIs. Groups that contained only one read (singletons) were merged into non‐singleton groups if the singleton's UMI could be converted to a UMI of a non‐singleton group by introducing an SNP (the biggest group was chosen).

### 2.9 Inflammatory Markers Expression Analysis

The analysis of 92 preselected inflammation‐related protein markers was conducted using the Olink Bioscience inflammation panel (Uppsala, Sweden) based on Proximity Extension Array (PEA) technology (Assarsson et al. 2014). Briefly, plasma samples were prepared by mixing 1 μL of plasma with a 3 μL incubation mix containing 92 pairs of specific probes, followed by overnight incubation at 4°C. Subsequent steps included the addition of an extension mix, a short incubation at room temperature, and a series of DNA extension and amplification cycles. Protein detection was facilitated by a 96.96 Dynamic Array IFC and analysed on a BioMark HD real‐time PCR system (Fluidigm). Results were expressed as Normalised Protein Expression (NPX) values, calculated on a log2 scale, after normalisation against control values to correct for technical and inter‐plate variations. The NPX values provide a relative quantification of protein concentrations, with higher NPX indicating higher protein levels. Quality control (QC) in the OLINK assay was maintained using control samples, inter‐plate controls for consistency across experiments, measurement of intra‐assay variability to ensure detection stability, and normalisation processes to adjust for variability in sample handling and assay conditions. This approach ensured the accuracy and reliability of the protein data collected. The limit of detection (LOD) was determined for each protein based on the mean value of triplicate negative controls analysed in each run. A missing data threshold of 10% was set because missing data beyond this level can negatively impact the quality, interpretability, and reliability of statistical analyses and avoid data imputation.

## 3 Data Analysis

The current study utilised the Data Integration Analysis for Biomarker discovery using Latent cOmponents (DIABLO) framework for integration of the different assessments. (Singh et al. 2019) all analyses were conducted using R studio (V. 2023.12.0), and data integration and DIABLO framework, a component of the mixOmics package (mixOmics V. 6.26.0) (Lê Cao et al. 2009; Rohart et al. 2017), was used.

DIABLO implements several approaches, such as multiblock partial least squares, generalised canonical correlation analysis, and multi‐omics factor analysis (Argelaguet et al. 2018; Li et al. 2012; Tenenhaus et al. 2014), and uses these in a supervised framework for selection and correlation of variables from multiple datasets into an interconnected network (Singh et al. 2019). DIABLO is also defined as multi‐blocks N‐integration, which requires the allocation of the different datasets in specific domains. In this study, four domains were included as follows: miRNAs expression levels, inflammatory markers expression, “QST”, and “PROMS” included evaluation of The Hospital Anxiety and Depression Scale for anxiety (HADS_A_) and depression (HADS_D_), Pain catastrophizing score (PCS), and Health‐related quality of life (H‐RQoL). Before analysis, data from each domain (miRNAs, inflammation, PROMS, and QST evaluations) underwent a preprocessing phase. This included scaling steps to ensure comparability across different data types, and variables with near‐zero variance were identified and removed to improve the model's performance and interpretability.

In the current study, the DIABLO model was used to explain if patients gained an analgesic effect from therapy. To do so, the analgesic effect of therapy (i.e., percentage change in KOOS pain scores compared to baseline and post‐therapy values) was calculated for each patient. After the calculation, two response groups were defined according to (Boonstra et al. 2016): Responder (≥ 30% analgesic effect) and Non‐responder (< 30% analgesic effect).

Partial Least Squares (PLS) pairwise comparisons were performed to identify the strength of the relationships between datasets, enabling the specification of design parameters that optimize component similarity and ensure robust data integration. The analysis was run with a design matrix of 0.55 value (from 0 to 1), indicating an assumption of partial interrelation among the various datasets. This was followed by the construction of a sparse Partial Least Squares Discriminant Analysis (sPLS‐DA) model, which actively excluded predictors with minimal variance. A tuning phase was run, where the optimal number of variables to retain in each component was determined. After fine‐tuning, the model incorporated the best combination of variables in the model.

### 3.1 Visualisation of Model

Various visualisations were generated, including Circos plots for cross‐correlations and loading plots to assess the relative contribution of each selected feature to group separation after DIABLO modelling. A visualisation of component 1 was chosen as this includes variables identified by the model that explains the most significant portion of the variation in the data. The Circos plot was configured with a correlation threshold of 0.8 to define the cross‐correlation between selected variables by the model, effectively identifying the pain mechanistic network. Loading plots were utilised to analyse variable relationships with component 1, classifying associations based on loading values into weak (−0.3 to 0.3), medium (0.3 to 0.7 or −0.3 to −0.7), and strong (> 0.7 or < −0.7). The visualisation was used for the interpretation of the discriminative features within the different data domains and for understanding the relationships between different data types.

### 3.2 Validation and Model Performance

The model tuning was executed within a robust ten‐fold cross‐validation framework (M‐fold (Hastie et al. 2009)), where in each iteration, nine folds served as the training set and one as the validation set. This approach ensured that the model parameters were optimized while mitigating the risk of overfitting. Following tuning, the model's performance was rigorously assessed using the same ten‐fold cross‐validation strategy. No independent validation dataset was used; thus, the reported metrics reflect internal cross‐validation performance.

To assess the model's discriminative ability, a confusion matrix was constructed to evaluate model performance. The confusion matrix tabulated the counts of true positives (TP), false positives (FP), true negatives (TN), and false negatives (FN) by comparing the model's predicted labels against the known ground truth. From these values, standard classification metrics were derived as follows: Sensitivity (True Positive Rate) = TP/(TP + FN); Specificity (True Negative Rate) = TN/(TN + FP); Accuracy = (TP + TN)/(TP + TN + FP + FN). To address the imbalance between outcome groups (77 non‐responders vs. 24 responders), model performance was evaluated using balanced accuracy and area under the precision‐recall curve (AUPRC) (Saito and Rehmsmeier 2015). Balanced accuracy was calculated as: Balanced Accuracy = Sensitivity + Specificity/2.

Sensitivity and specificity were derived from the confusion matrix, and 95% confidence intervals for balanced accuracy were estimated using standard error propagation. For AUPRC, predicted class probabilities for the responder group were averaged across blocks and used as input to compute precision‐recall curves. AUPRC and corresponding 95% confidence intervals were estimated using 1000 bootstrap iterations implemented in the precrec R package (V 0.14.5). Precision‐recall curves were visualised using ggplot2. These metrics allowed for a direct quantification of the model's classification performance in terms of its ability to correctly identify positive cases, correctly reject negative cases, and produce an accurate overall prediction.

## 4 Results

### 4.1 Demographics

One hundred one patients (53 female) with a mean age of 63.6 (CI: 61.6–65.6) years and a mean body mass index of 28.9 (CI: 27.9–30.0) kg/m^2^ participated. The mean KOOS pain scores were 51.7 (CI: 48.4–55.0) at baseline and 60.1 (CI: 56.7–63.6) at follow‐up, reported to be significantly different (*p* < 0.001). Patients were divided into two groups, including 77 patients in the non‐responder (< 30% analgesic effect) group and 24 in the responder group (≥ 30% analgesic effect). No adverse events were reported during the study.

### 4.2 Detection of microRNAs and Inflammatory Markers

For miRNAs, the mapping rates, defined as the percentage of sequencing reads aligning to known miRNAs, varied from 24% to 78%, indicating a low to moderate matching to sequence references, defined as known miRNA sequences in the reference database, which was sufficient for all downstream analyses. Analysis of reads mapped with miRBase revealed the presence of 2633 miRNA species in the evaluated samples. MiRNAs mapped that were not expressed in at least 50% of the patients were excluded due to their contribution to the variance, and the remaining 1110 miRNAs were included in the subsequent analysis.

Similarly, for inflammatory markers analysis, the proximity extension array approach allowed the identification of 68 markers above the limit of detection. Moreover, before the set‐up of the model, inflammatory markers contributing zero to the variance were excluded. The final analysis resulted in including 68 markers in the final model.

### 4.3 Data Integration and DIABLO Modelling

**FIGURE 1:**
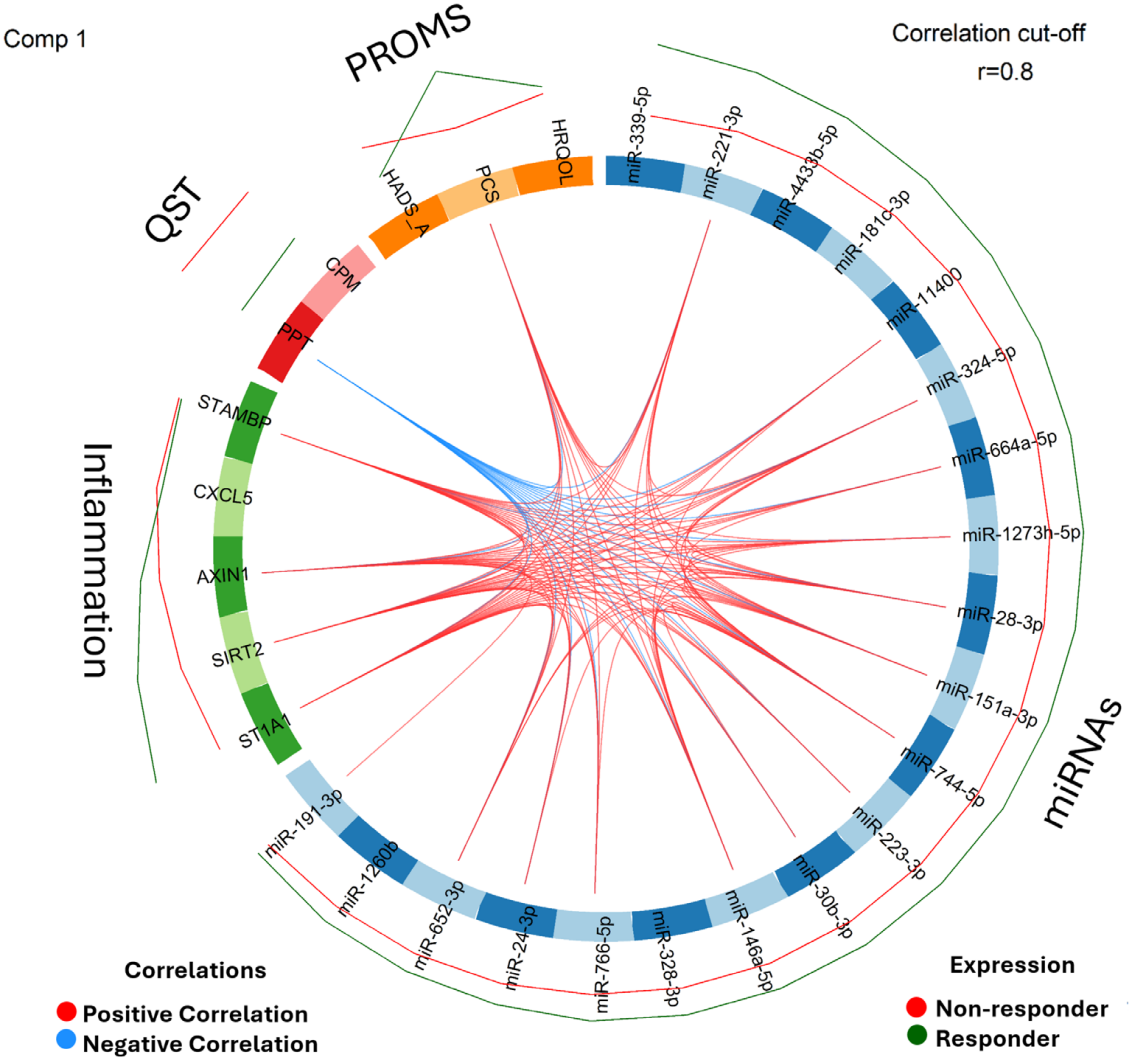
Circos plot from DIABLO modelling for component 1. The plot represents the correlations greater than 0.8 between variables selected for all domains. The internal connecting lines show the positive (red) and negative (blue) inter‐correlations. The outer lines show the expression levels of each variable in each group, non‐responder (red), and responder (dark green).

The integration of patients' data for every data domain (miRNAs, Inflammation, QST, PROMS) was investigated between the responder and non‐responder groups. Individuals for each domain were not distinctly separated, indicating a degree of overlap or intermixing among the groups when single domains were evaluated with PLS‐DA (S1). The tuning of the DIABLO model highlighted 30 variables related to group categorization as the best combination of variables for the model, which included 20 miRNAs, 5 inflammatory markers, PPT and CPM for the QST domain, and quality of life, pain catastrophizing, and anxiety for the PROMS domain. Raw values for all variables in the final feature set are presented as individual data points across the two outcome groups (responders vs. non‐responders) to provide transparency and facilitate interpretation (Figures S2–S5). The Circos plot illustrates the cross‐correlations between the 30 variables included in the model, with variables highly correlated with each other (Pearson's correlation coefficients > 0.8, see Figure 1). Moreover, single variables included in the model showed a relative alteration between the two groups (outer ring in Figure 1).

**FIGURE 2:**
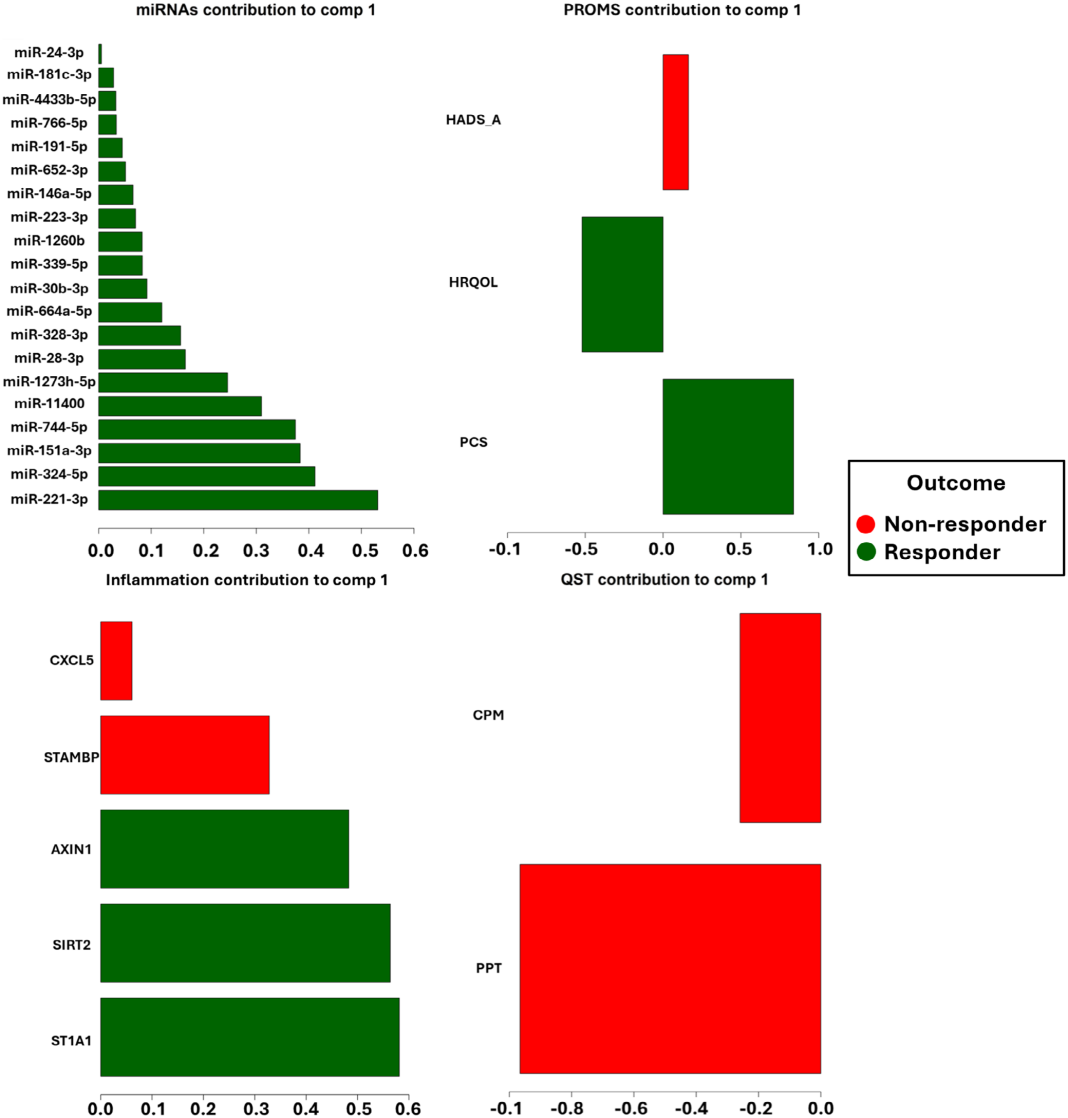
Loading plot for variables of each domain and their contribution for component 1. The loading plot depicts of the most important variables ordered from bottom to top. The absolute value of the loading score indicates the importance or contribution of a variable to component 1. Colours indicate the class for which the average expression value is the highest for each feature responder (dark green), non‐responder (red).

Furthermore, the loadings plot from the final DIABLO model revealed distinct weight contributions of the selected variables, highlighting those with the strongest influence on the discrimination between responders and non‐responders. MiRNA loading values (Figure 2) reported a moderate to strong association with the first component. The 20 miRNAs defined by the model in this block showed a positive weight for the responder group outcome.

The protein variables presented loading values indicating a moderate to strong positive weight association with component 1. Specifically, variables AXIN1, SIRT2, and ST1A1 showed a positive weight for the responder group, whereas CXCL5 and STAMP markers reported a weak to moderate positive weight for the no relief group (Figure 2). QST variables, CPM, and PPT showed a weak negative weight for the non‐responder groups (Figure 2). Among the PROMS variables chosen by the model, H‐RQoL demonstrated a weak negative weight for the responder group and PCS showed a weak positive weight for the same group. The HADS_A_ showed a weak negative weight for the non‐responder group (Figure 2).

### 4.4 Performance of the Model to Predict Analgesic Effect

The model yielded a sensitivity of 83% and specificity of 87%, corresponding to an overall accuracy of 84% (95% CI: 0.75–0.91). Given the class imbalance (77 non‐responders vs. 24 responders), performance was more appropriately summarized by a balanced accuracy of 0.853 (95% CI: 0.770–0.937). For the responder class, the area under the AUPRC was 0.858 (95% CI: 0.636–0.971).

## 5 Discussion

The current study demonstrated that it is possible to predict the analgesic effect (above or below 30% analgesic effect) of a combined anti‐inflammatory drug with paracetamol in knee OA with a sensitivity of 83%, a specificity of 87%, and a balanced accuracy of 85% by integrating pain sensitivity, inflammation, microRNA, and PROMS. No previous method has predicted the effect of pharmacological intervention with such high sensitivity.

### 5.1 Pain Sensitivity

QST is a widely used assessment tool for evaluating pain sensitivity, including proxies for pain sensitization (Dyck et al. 1993; Rolke et al. 2006; Wilder‐Smith 2013). Several studies have consistently demonstrated an association between increased pain hypersensitivity and higher clinical pain (Arendt‐Nielsen, Skou, et al. 2015; Arendt‐Nielsen et al. 2018; Petersen 2022; Petersen et al. 2023). Additionally, studies demonstrated that patients characterized by increased pain sensitivity showed a higher risk for developing persistent postoperative pain (Izumi et al. 2017; Kurien et al. 2018; Petersen et al. 2018), lowered analgesic effect of NSAID‐based pharmacological therapies (Arendt‐Nielsen et al. 2016; Petersen, Olesen, et al. 2019; Petersen, Simonsen, et al. 2019), and other analgesic medications, such as duloxetine (Petersen et al. 2022). Furthermore, the tools have been shown to quantify the effectiveness of non‐pharmacological and non‐surgical interventions (Hansen et al. 2020; Lyng et al. 2022; Petersen et al. 2020). In the current study, QST was important for differentiating responders from non‐responders. Pressure pain threshold demonstrated strong negative correlations with inflammatory markers and miRNAs expression levels. These findings suggest a potential interaction between sensitivity and inflammatory or epigenetic mechanisms in patients with knee OA, and the results of DIABLO support the concept that pain mechanisms, assessed via QST, together with other factors, can predict pharmacological responses in knee OA patients.

### 5.2 Psychological Factors

Psychological factors such as anxiety, depression, and pain catastrophizing have all been consistently associated with chronic pain (Edwards et al. 2011). Psychological factors are likewise recognised as predictors of poor therapeutic outcomes in musculoskeletal pain intervention trials (Edwards et al. 2011; Larsen et al. 2021). Previous studies have demonstrated associations between elevated pain catastrophizing, anxiety, and depression scores, and increased likelihood of chronic postoperative pain, as well as poorer responses to pharmacological and exercise‐based interventions (Larsen et al. 2021; Petersen et al. 2022). Furthermore, a previous study, which analysed the same cohort of knee OA patients using less advanced methods, demonstrated that pain catastrophizing is a strong mediator of pain in osteoarthritis, with a greater contribution than that observed through QST (Hertel et al. 2024). However, psychological factors and QST independently contributed to clinical pain severity, emphasising the importance of assessing both psychological and sensory domains when evaluating pain conditions (Hertel et al. 2024). The current study further supports this by demonstrating that a predictive model combining pain catastrophizing, anxiety, and quality of life effectively distinguishes between knee OA patients who respond and do not respond to pharmacological therapy.

### 5.3 Inflammation

Among biological factors, inflammation is recognised as a major contributor to pain in OA (Giordano, Ghafouri, et al. 2024; Schaible 2014). Inflammatory markers such as interleukin‐1β (IL‐1β), interleukin‐6 (IL‐6), and tumour necrosis factor‐alpha (TNF‐α) have been consistently found to be elevated in patients with musculoskeletal pain and are positively associated with increased clinical pain severity (Eitner et al. 2017; Gandhi et al. 2013).

In the present study, inflammatory markers including CXCL5, STAMBP, AXIN1, and SIRT2 were included in the final DIABLO model as markers important for the separation of responders and non‐responders to therapy. A recent study has evaluated the involvement of CXCL5 for pain, showing how serum levels of this marker are upregulated in male patients with gouty arthritis (Yin et al. 2024). Moreover, as demonstrated in animal models, the regulation of the CXCL5 and CXCR2‐TRPA1 axis contributes to nociceptive activity in arthritis (Yin et al. 2024). STAMBP is an enzyme involved in the JAK–STAT signal transduction pathway, playing a role in the inflammatory response and the promotion of pro‐inflammatory cytokines (Bednash et al. 2017). Previous research has demonstrated that this pathway, through regulation and active induction of pro‐inflammatory cytokine release, is implicated in the pathogenesis of rheumatoid arthritis (RA) (Simon et al. 2021). The remaining markers, including AXIN1, SIRT, and especially ST1A1, have been suggested to have protective effects against osteoarthritis and may serve as potential biomarkers for pain (Karshikoff et al. 2023; Lin et al. 2013). The findings from the present study align with previous research, providing further evidence that may clarify the mechanisms underlying chronic pain and help explain how these markers contribute to therapeutic responses.

### 5.4 Epigenetics Modifications

Epigenetic mechanisms have in recent years been suggested to be implicated in the development and maintenance of chronic pain (Mauceri 2022). Among epigenetic modifications, miRNAs have been shown in several studies to have an impact on pain in general, including knee OA–related pain (Li et al. 2024; Polli et al. 2020). Human studies have shown how specific miRNAs might represent risk factors for OA, influence its severity, and regulate the expression of targets used in therapies (Li et al. 2024). The research mentioned supports the idea that miRNAs may serve not only as a signature for pain but also as potential biomarkers for personalised pain management. The most weighted miRNAs, which were able to differentiate responders and non‐responders in this study, have recently been shown to be involved in pain in OA (Giordano et al. 2020). For instance, Woods et al. report how miR‐324‐5p was upregulated in the end stage of OA in humans, leading to cartilage disruption (Woods et al. 2019). Moreover, preclinical studies have demonstrated how miRNAs such as 221 and 151a have opposite effects in inducing and sustaining neuropathic pain, via regulation of key genes involved in signal transduction and cytokine activation (Xia et al. 2016; Zhang et al. 2021). In the present study, a combination of 20 miRNAs, including miRNAs previously associated with pain and OA pathophysiology, such as miR‐146a‐5p and miR‐30b (Giordano et al. 2020), was identified within the final predictive model, further supporting the significant contribution of these epigenetic markers in predicting therapeutic response.

### 5.5 Biomarker Performance in Osteoarthritis and Pain Prediction

Sensitivity, specificity, accuracy, and the Area Under the Curve (AUC) are critical measures for assessing the performance of diagnostic biomarkers and predictive models, and in a recent study employing deep learning‐based clustering using knee OA multi‐omics data, an integrative multimodal approach combining data from markers evaluated in plasma, synovial fluid, and urine significantly improved predictive performance for postsurgical pain and function outcomes, achieving integrated model AUCs ranging from 0.74 to 0.88 (Rockel et al. 2025). Similarly, in chronic pain modelling, cortical biomarker signatures achieved an AUC of 100% (validation set) and 88% (test set), reflecting high predictive accuracy using distinct neural oscillatory and corticomotor excitability measures (Chowdhury et al. 2025). Thus, the sensitivity, specificity, accuracy, and AUC values reported in this study align with the higher performance ranges observed in existing literature, supporting the robust diagnostic potential of our approach for clinical utility in OA management.

### 5.6 Limitations

The current study is a secondary analysis, and a predetermined sample size calculation was not performed. Sample size calculation for the DIABLO models is complex, as the number of samples should be evaluated for each domain and the variables included within it. A post hoc sample size estimation, based on multiple regression models, which provides a closer comparison to the DIABLO model, suggests that our model can identify 34 parameters with 80% power, a significance level of 0.05, and a large effect size, with a sample size of 101 patients.

Our model identified 30 parameters, and therefore, the current analysis might not be underpowered. In addition, model performance was evaluated using 10‐fold cross‐validation during both tuning and performance estimation (Singh et al. 2019), without the use of an independent validation dataset. While cross‐validation reduces the risk of overfitting compared to using a single train‐test division, it does not fully eliminate overestimation when applied to unseen data. Therefore, the reported performance metrics should be interpreted with caution, and future studies should confirm these findings using an independent validation cohort to ensure generalizability. Our analysis was able to reduce the final model to 30 parameters. This is an improvement when compared to the 1184 parameters that were included in the original model, but this is not yet at a level that can be implemented in the clinic. Future work should focus on further reducing the number of parameters so that the results can be utilized in the clinic. Several relevant factors were not assessed in this study. For example, hormonal factors in females (e.g., oestrogen levels and menopausal status) can influence pain sensitivity and inflammation in osteoarthritis (Wright et al. 2024). Likewise, lifestyle habits such as diet, physical activity, sleep, and stress, which affect pain, inflammation, and epigenetic processes (González‐Rodríguez et al. 2023; Nijs et al. 2020), were not systematically evaluated. Including these factors in future research could strengthen the associations explored in this work. Lastly, only one machine‐learning approach (DIABLO) was applied. DIABLO was selected because it is particularly well suited for integrating multi‐omics and clinical data, offering interpretability and robustness (Singh et al. 2019). Nevertheless, the use of additional algorithms could provide a more comprehensive assessment of predictive performance. Future research should compare multiple models to ensure the robustness of the observed patterns.

## 6 Conclusion

The current study utilises the DIABLO model to predict the analgesic effect of anti‐inflammatory drugs and paracetamol therapy in patients with knee osteoarthritis. Our model demonstrates that measures of pain sensitivity, psychological factors, miRNAs, and inflammation are interconnected and can form networks predicting therapy outcome with a sensitivity of 83%, a specificity of 87%, and a balanced accuracy of 85%. This work corroborates the use of complex data and their analysis through machine‐learning techniques with a holistic approach, which may be the new avenue towards personalised pain management.

## Author Contributions

L.A.‐N., A.E.O. and K.K.‐S.P. designed the study, R.G. conducted the experimental inflammatory analysis, the microRNA analysis, and conducted the statistical analyses; R.G., L.A.‐N., A.E.O., E.H. and K.K.‐S.P. contributed to the interpretation of data and drafted the manuscript. All authors critically reviewed the manuscript and approved the final version of the manuscript before submission.

## Conflicts of Interest

The authors declare no conflicts of interest.

## Supporting information


**Data S1:** Supporting Information.

## Data Availability

Data relative to this work will be available upon reasonable request to the corresponding author.
