# Supplementary material for: Multifactorial Machine Learning Algorithm Integration of Pain Mechanisms Can Predict the Efficacy of 3‐Week NSAID Plus Paracetamol in Patients With Painful Knee Osteoarthritis
Source: Eur J Pain. 2025 Sep 29;29(10):e70140. doi: 10.1002/ejp.70140 (PMC12478294; doi:10.1002/ejp.70140)

# Supplementary Materials

**S1.**  Partial Least Squares Discriminant Analysis (PLS-DA) sample plot from DIABLO modeling. Single samples are plotted based on their scores on the first two latent components (Component 1 and Component 2) for each domain. Samples are colored by the non-responder (red circles) and responder (dark green triangles) groups. Ovals depict the 95% confidence intervals.


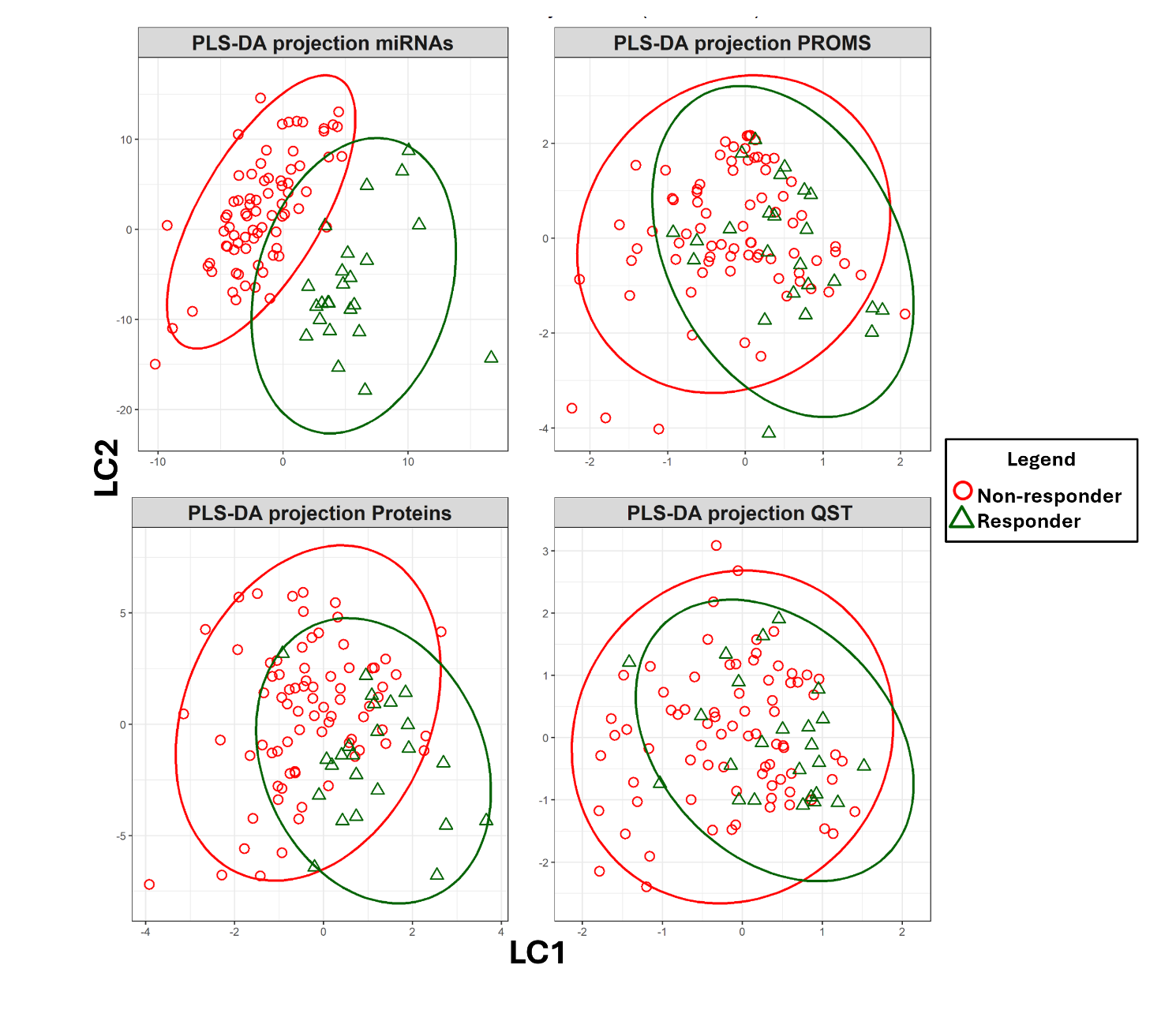


**S2. Raw expression of selected miRNAs.** Individual raw data points for the 20 miRNAs included in the final feature set, shown across outcome groups (no relief vs. relief). Jittered scatter plots are displayed for each miRNA, with values normalized to internal controls. Colors represent the outcome group (red = no relief, green = relief).


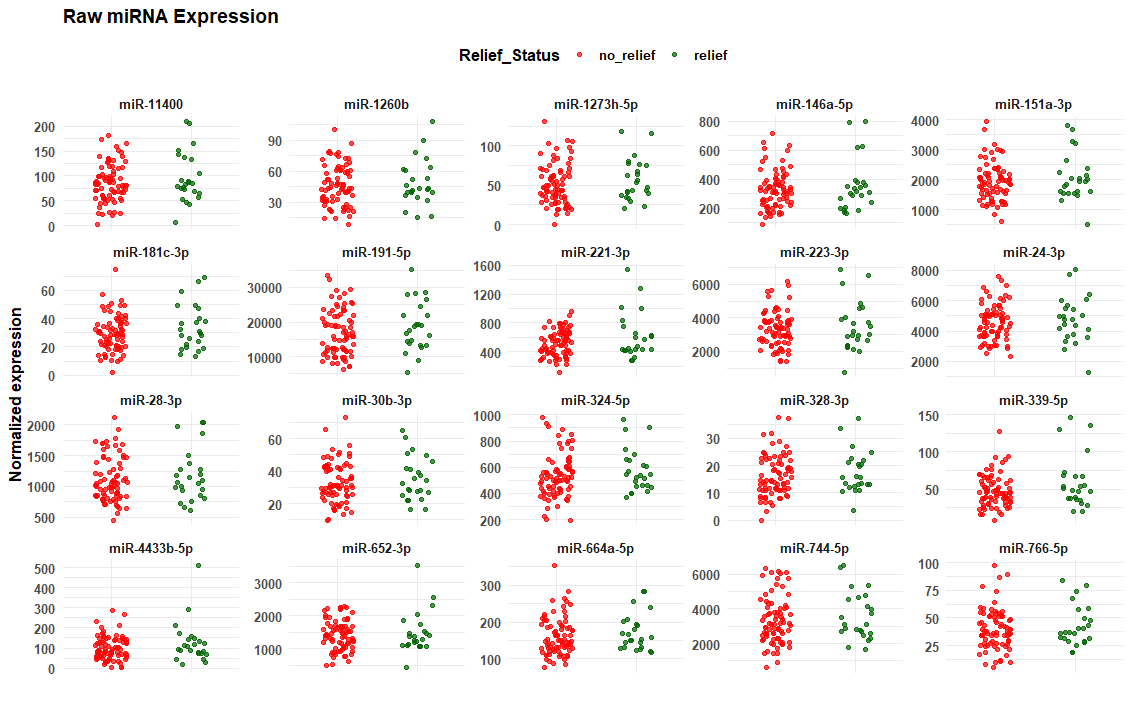


**S3. Raw PROMs scores**. Individual raw data points for the three selected patient-reported outcome measures (HADS-A, HRQOL, PCS), shown across outcome groups (no relief vs. relief). Colors represent the outcome group (red = no relief, green = relief).
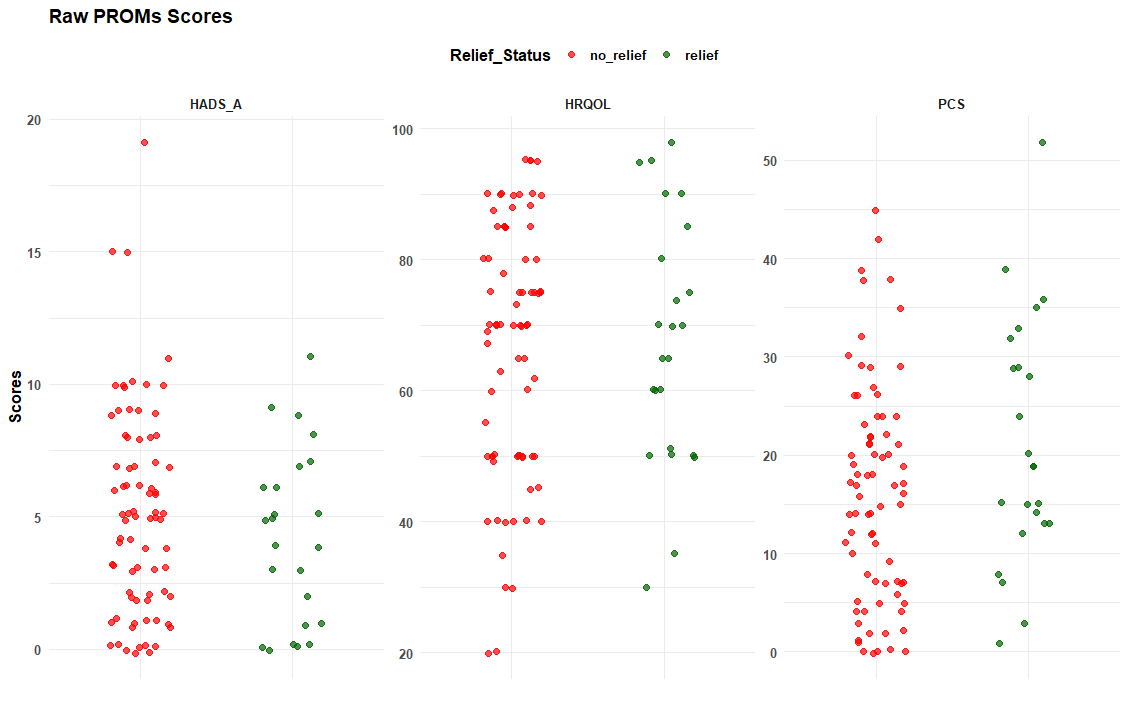


**S4. Raw expression of selected markers of inflammation**. Individual raw data points for the five selected proteins (CXCL5, STAMBP, AXIN1, SIRT2, ST1A1) included in the final feature set, shown across outcome groups (no relief vs. relief). Data are presented in NPX units (normalized protein expression). Colors represent the outcome group (red = no relief, green = relief).
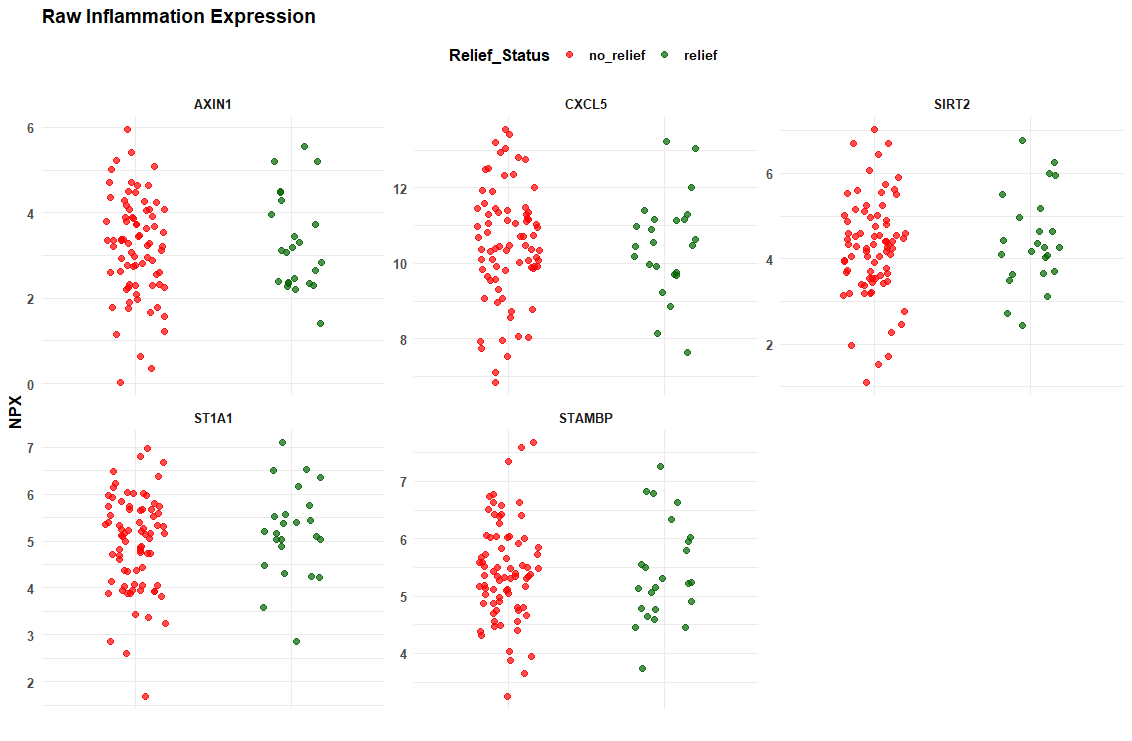


**S5. Raw QST measures.** Individual raw data points for the two selected QST measures (CPM, PPT), shown across outcome groups (no relief vs. relief). Colors represent the outcome group (red = no relief, green = relief).


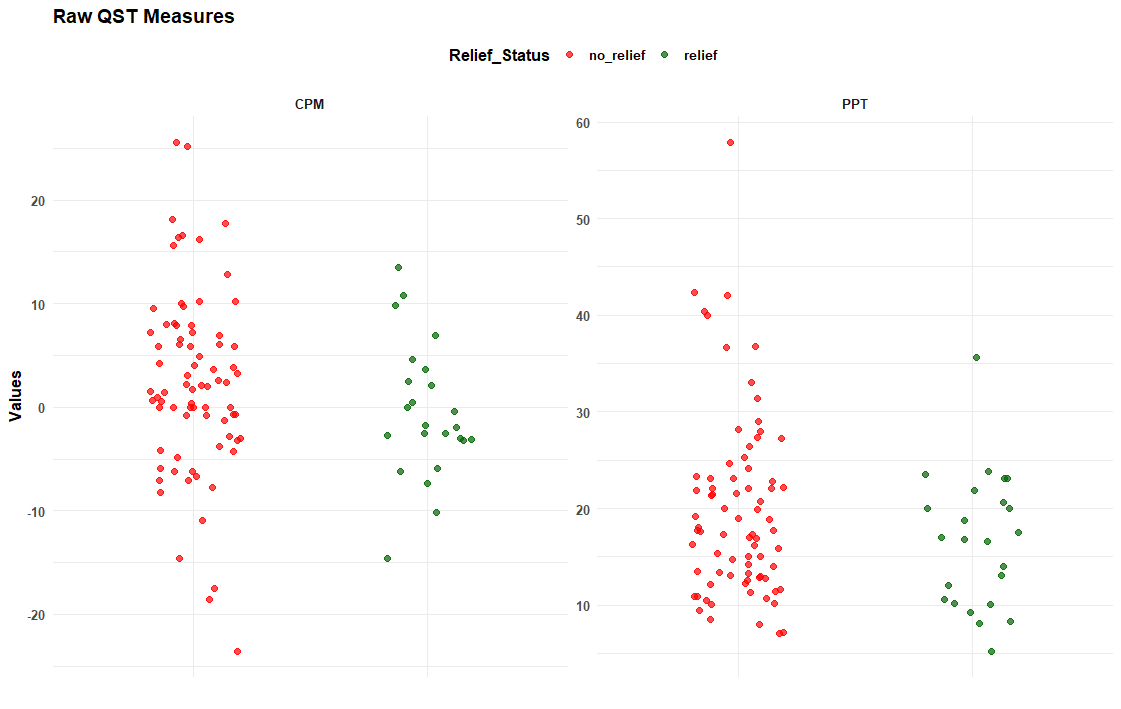

Supplement: Supplementary file 1 — Data S1: Supporting Information. [file EJP-29-0-s001.docx]
